# The Relationship between Metal Exposure and Chronic Obstructive Pulmonary Disease in the General US Population: NHANES 2015–2016

**DOI:** 10.3390/ijerph19042085

**Published:** 2022-02-13

**Authors:** Qiaoyuan Fei, Xueqiong Weng, Kun Liu, Shan Liu, Jingmin Chen, Xinrong Guo, Chunxia Jing

**Affiliations:** 1 Department of Public Health and Preventive Medicine, School of Medicine, Jinan University, No.601 6 Huangpu Ave West, Guangzhou 510632, China; qy824559259@163.com (Q.F.); wengxueqiong111@gmail.com (X.W.); liukungyyf@163.com (K.L.); lshliushan@163.com (S.L.); canotwwwwait@stu2019.jnu.edu.cn (J.C.); abbyla@foxmail.com (X.G.); 2 Guangdong Key Laboratory of Environmental Exposure and Health, Jinan University, Guangzhou 510632, China

**Keywords:** metals, trace minerals, COPD, RCS, copper

## Abstract

The effects of metal on pulmonary function are inconsistent, and abnormal distribution of metals can decrease lung function. However, the effects of metals exposure on chronic obstructive pulmonary disease (COPD) are still unclear. This study aims to explore the relationship between metal exposure and COPD risk. Cross-sectional data from the National Health and Nutrition Survey (NHANES) 2015–2016 was analyzed. Inductively coupled plasma dynamic reaction cell mass spectrometry (ICP-DRC-MS) was used to measure the metals concentration in the blood. The multiple linear regression and restricted cubic spline (RCS) were used to analyze the relationship between metals exposure and COPD risk. In this study, 1399 participants were included, of which 107 participants were diagnosed with COPD using self-reported chronic bronchitis, emphysema, and COPD. The second and third tertiles of copper increased the COPD risk by 1.98-fold (95% CI: 1.08–3.62) and 2.43-fold (95% CI: 1.32–4.48) compared with the first tertile, using *p* = 0.005 for the trend after adjusting for the covariates. RCS showed a positive linear correlation between copper and COPD risk (*p* = 0.006 for overall association) in all participants. When stratified by sex, the multi-factor analysis showed that the third tertile of copper increased male’s COPD risk by 3.42-fold (95% CI: 1.52–7.76), with *p* = 0.003 for the trend, and RCS also showed a positive linear correlation (*p* = 0.013 for overall association). Although RCS showed that selenium can reduce the COPD risk (*p* = 0.008 for overall association) in males, an association between selenium and COPD was not observed (*p* > 0.05). Our findings suggest that a high concentration of copper may increase COPD risk in males in the general US population, and more research is needed to explore its possible mechanism of action.

## 1. Introduction

Chronic obstructive pulmonary disease (COPD) is a disease characterized by persistent airflow limitation caused by large amounts of exposure to toxic particles and gases. It includes chronic bronchial and emphysema [1]. The most common symptoms of COPD are dyspnea, cough, and sputum production [2]. According to statistics from the World Health Organization (WHO), there are currently about 600 million people suffering from COPD in the world, and an average of about 2.7 million people die from COPD every year [3]. Due to the high prevalence, morbidity, and mortality of COPD, it has brought a great burden of disease to the world, it has always been one of our main public health issues [4]. COPD ranked 11th among the top 15 major diseases that caused the loss of disability-adjusted healthy life years in 1990, and by 2019, COPD rose to 6th. Additionally, 66% of the global burden of COPD and lung cancer comes from low- and middle-income countries [5]. Smoking, indoor air pollution (such as biofuels used for cooking and heating), outdoor air pollution, occupational dust, and chemicals are the main risk factors for COPD, among which smoking is the most important factor [6,7,8]. Therefore, the first measure to prevent and treat COPD is to quit smoking. In addition, vaccinations and drugs can also be used to prevent and treat COPD [2].

Metals may have different effects on pulmonary function. Copper, zinc, and selenium are essential trace elements for the human body. Copper in tap water was positively related to levels of both FVC and FEV1 among never smokers [9]. Xiao, Zhou et al. reported that copper can reduce the forced expiratory volume in 1 s [10]. Cadmium can enter the human body with tobacco drugs and can accumulate in the body, especially in the lungs [11,12], and the accumulation of cadmium can cause lung inflammation and a decline in lung function [13,14,15]. Zinc has an antagonistic effect on the toxicity of cadmium and copper [13,16]. A lack of zinc may lead to impaired immune function and tumor development [17,18,19]. An increased prevalence of obstructive lung disorder was observed among individuals with low zinc intake regardless of smoking status, and the adjusted odds of lung disorder are approximately 1.9 times greater for subjects in the lowest zinc-intake tertile than those in the highest tertile (odds ratio = 1.89, 95% confidence interval = 1.22–2.93) [20]. Selenium is related to the cellular antioxidant defense mechanism. Oxidative stress and chronic inflammation are important features in the pathogenesis of COPD [21]. However, the relationship between selenium and COPD is unclear. Studies have shown that the accumulation of manganese reduces the expression of cystic fibrosis transmembrane conductance regulator (CFTR), which is a chloride channel located in airway epithelial cells. CFTR plays an important role in maintaining the dynamic balance of airway surface fluid (ASL) volume and the physiological function of the lungs. If the expression decreases or the function is impaired, the lungs cause mucus accumulation, reduced bacterial clearance, and chronic infection and inflammation [22]. A study in NHANES 2007–2010 found a dose-dependent relationship between blood lead concentration and the risk of COPD [23].

Since the associations between metal exposure and trace minerals, and COPD remain unclear, and nothing is known about their dose–response relationship, we used the data from NHANES (2015–2016) to explore the relationship between metal and trace minerals, and COPD.

## 2. Materials and Methods

### 2.1. Study Population

The National Health and Nutrition Examination Survey (NHANES) is a cross-sectional survey based on the whole population of the United States. The study protocol was approved by the NCHS Research Ethics Review Board (Continuation of Protocol #2011-17), and all participants provided written informed consent. It combines questionnaire surveys and physical examinations to collect information about the health and nutrition of the American family population, including demographics, diet-related issues, physical examinations, laboratory examinations, and more [11].

**Figure 1:**
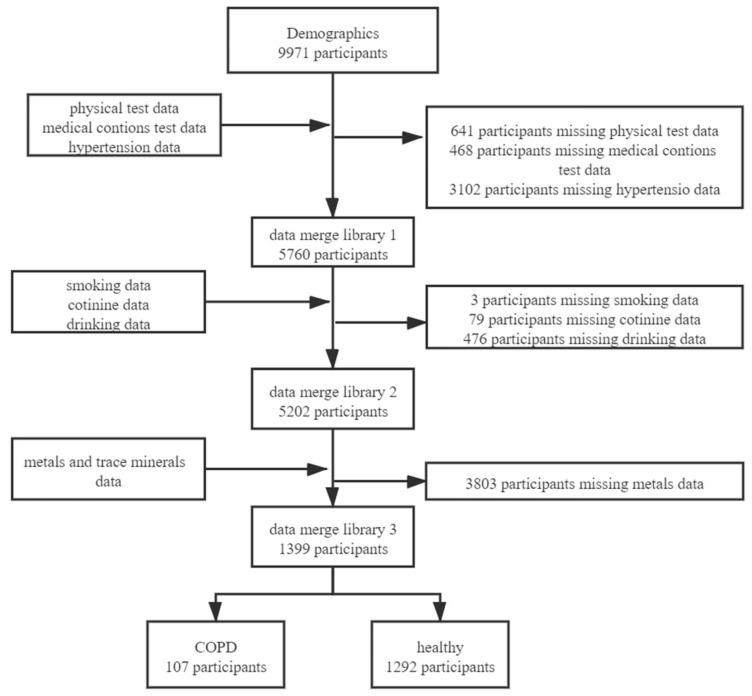
Flowchart for the inclusion of study participants.

The data from NHANES 2015–2016 was analyzed, and the 9971 participants were screened. We merged the databases based on the unique identity of the survey subjects. After merging the databases, we excluded 8572 who had missing data in physical examination, medical conditions, drinking, smoking, second-hand smoke exposure, serum cotinine, hypertension, diabetes, and blood metal measurements. Finally, 1399 survey subjects were included in the study, including 694 males and 705 females (Figure 1).

### 2.2. Metal and Trace Mineral Measurements

The processed blood were stored under −20 °C conditions and sent to the laboratory for analysis. The levels of metals were measured using inductively coupled plasma dynamic reaction cell mass spectrometry (ICP-DRC-MS) by the Centers for Disease Control and Prevention in the US [24,25,26].

### 2.3. COPD

Current chronic bronchitis was defined by positive answers to the questions (1) “Has a doctor or other health professional ever told you that you have chronic bronchitis?” and (2) “Do you still have chronic bronchitis?” Emphysema was defined by positive answers to the questions: “Has a doctor or other health professional ever told you that you have emphysema?” COPD was defined by positive answers to the questions: “Has a doctor or other health professional ever told you that you have COPD?” [1].

### 2.4. Smoking and Secondhand Smoke Exposure

Smokers were divided into three groups: non-smokers, current smokers, and former smokers. Non-smokers were defined as smoking less than 100 cigarettes in their lifetime. Current smokers were defined as smoking more than 100 cigarettes in their lifetime and were still smoking. Former smokers were defined as smoking more than 100 cigarettes in their lifetime but not smoking now [20,27].

Participants who had been to jobs, bars, restaurants, other homes, and other indoor places and who had smoked cigarettes in these places were included in the secondhand smoke exposure group.

### 2.5. Drinking

Participants were divided into drinking groups and non-drinking groups according to whether they had drunk more than 12 alcoholic drinks in their lifetime [28].

### 2.6. Covariates

We included the covariates based on previous studies [3,29], which might be related to COPD or the concentration of metal. We collected basic information about participants through questionnaires, including gender; age; race (Mexican American, other Hispanic, non-Hispanic white, non-Hispanic black, and other race); education (less than 9th grade, 9–11 grade, high school graduate, some college/AA degree, and college graduate); the ratio of family income to poverty and BMI (<18.5, 18.5–25, 25–30, ≥30); and hypertension and diabetes, which might be related to COPD or the concentration of metal [3,29].

### 2.7. Statistical Analysis

All analyses were performed with Stata version 15.0 (StataCorp LP, College Station, Texas, USA) and R software version 4.1.0 (R Foundation for Statistical Computing, Vienna, Austria). RCS was implemented with the R package “rms” (version 6.1-1) (Frank Harrell, Nashville, Tennessee, USA). The continuous variables were represented by mean ± SD, non-normally distributed continuous variables were represented by the interquartile range (IQR), and categorical variables were represented by cases (n) and percentage (%). The Chi-square test was used to compare the demographic differences between the COPD cases and the control group, including age, gender, smoking, PIR, BMI, race, education, alcohol consumption, second-hand smoke exposure, high blood pressure, and diabetes. As the distributions of metals were right-skewed (Figure S1), the heavy metals were standardized via a natural ln (loge) transformation and the Wilcoxon rank-sum test was used to compare the metal concentration between the case group and the control group. A multiple logistic regression model was used to analyze the relationship between metals exposure and the risk of COPD. The metal exposure level was divided into tertiles (T1, T2, and T3 were the first, second, and third tertiles, respectively), T1 was used as a reference, and odds ratios (ORs) and 95% confidence intervals (95% CIs) were used to describe the relationship between metal exposure levels and diseases. RCS [30] was used to further analyze the relationship and trend between metal exposure and COPD because RCS not only can analyze the linear relationship between metals and the risk of COPD but also can reflect the nonlinear relationship between the two.

## 3. Results

### 3.1. Demographic Characteristics

In the study, 1399 participants were included; 107 participants with emphysema, chronic bronchitis, and COPD were classified as the COPD group; and 1292 were classified as the healthy group. There was a difference in the age distribution between the COPD group (58.53 ± 16.19) and the healthy group (48.81 ± 17.43) (*p* < 0.001). The results showed a significant difference in smoking between the two groups (*p* < 0.001). The proportion of former smoking in the COPD group (35.45%) was higher than that in the healthy group (23.08%). The COPD group had higher levels of serum cotinine (115.13 ± 166.73) than the control group (46.26 ± 108.12) (*p* < 0.001). Significant differences in race, BMI, education, the ratio of family income to poverty, secondhand smoke exposure, hypertension, and diabetes (*p* < 0.05) were also observed. The levels of lead, calcium, and copper in the COPD group were higher than those in the healthy group (*p* < 0.05). However, the levels of selenium in the COPD group were lower than in the healthy group (*p* < 0.05) (Table 1).

### 3.2. The Association between Metals, Trace Mineral Exposure, and the Risk of COPD

**Figure 2:**
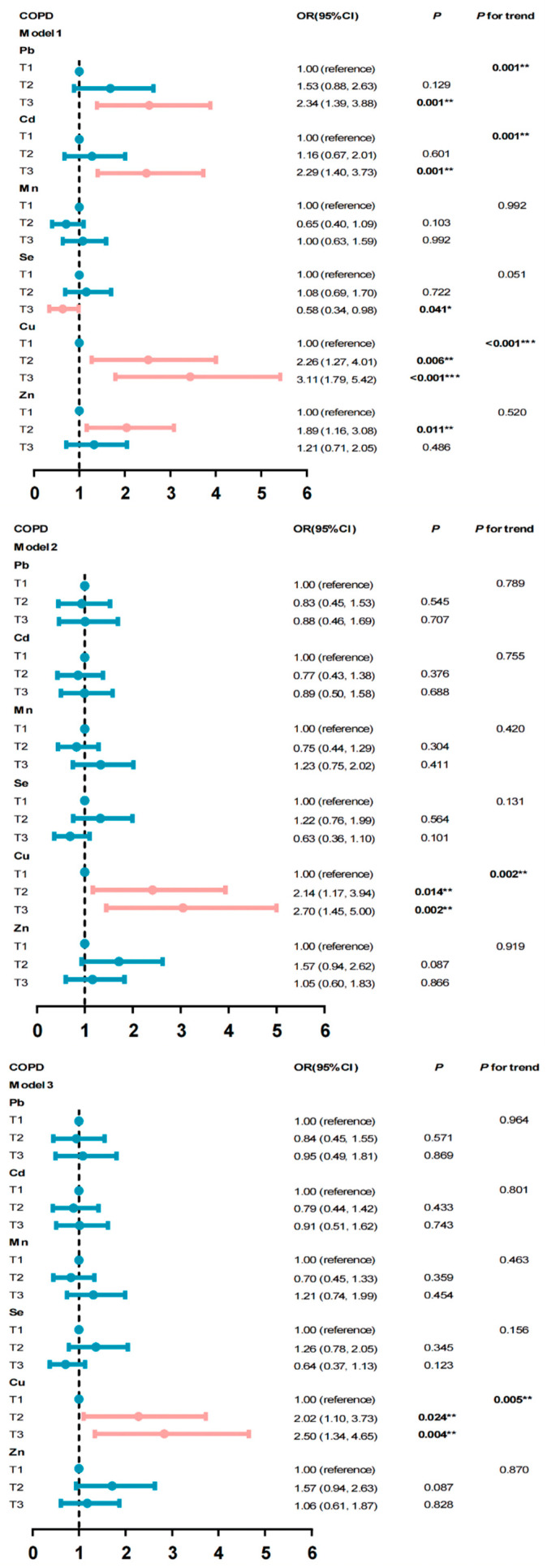
Association between COPD and metals. Model 1: unadjusted model; Model 2: adjusted for age, smoke, ratio of family income to poverty, BMI, sex, race, education, drink, second-hand smoke exposure, and serum cotinine; Model 3: adjusted for age, smoke, ratio of family income to poverty, BMI, sex, race education, drink, second-hand smoke exposure, serum cotinine, hypertension, and diabetes. * *p* < 0.05; ** *p* < 0.01; and *** *p* < 0.001.

The second and third tertiles of copper increased the risk of COPD by 2.02 times and 2.50 times compared with the first tertile after adjusting for other covariates, respectively, the *p*-value for trend was 0.005 (T2 95% CI: 1.10–3.73; T3 95% CI: 1.34–4.65) (Figure 2). We further analyzed their relationships stratified by gender. The third tertile of copper only increased COPD risk in males by 3.31 times (*p* = 0.004, 95% CI: 1.47–7.44) compared with the lowest tertile, and the *p*-value for trend was 0.004 (Table 2).

### 3.3. Dose–Response Relationship between Metals and the Risk of COPD

**Figure 3:**
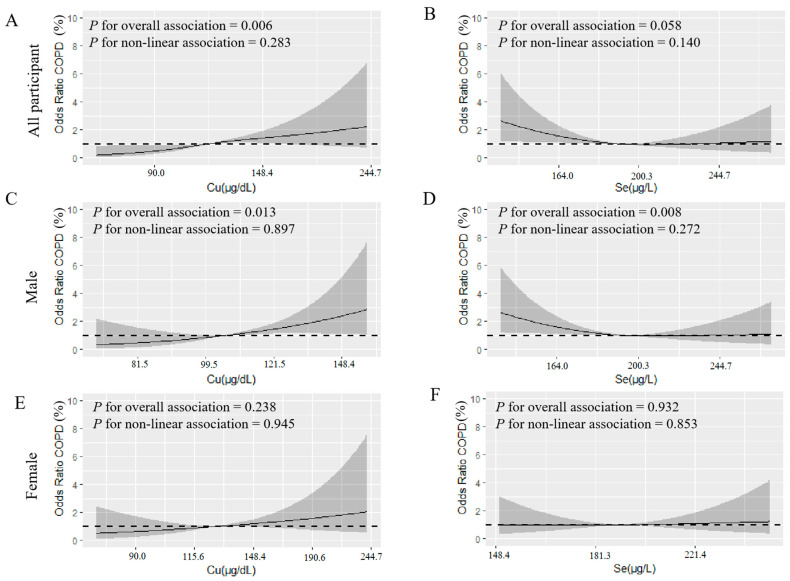
(**A**–**F**) used restricted cubic spline modeling to analyze the relationship between metals, trace minerals, and COPD (ln-transformed). (**A**,**B**) is the relationship between copper, selenium, and COPD in all survey subjects, (**C**,**D**) is the relationship between copper, selenium, and COPD in males, and (**E**,**F**) is the relationship between copper, selenium, and COPD in females. The analysis adjusted for age, smoke, ratio of family income to poverty, BMI, sex, race education, drink, second-hand smoke exposure, serum cotinine, hypertension, and diabetes. The black solid line represents the combined restricted cubic spline curve model, and the shaded part represents the 95% CI of the combined curve.

RCS showed that there was a linear relationship between ln(copper)- exposure and the risk of COPD (*p* = 0.006 for overall association) in all participants. As the ln(copper) level increased, the risk of COPD increased (Figure 3A). Both ln(copper) (*p* = 0.013 for overall association) and ln(selenium) (*p* = 0.008 for overall association) had linear relationships with the risk of COPD in males. As the ln(copper) level increased, the risk of COPD increased (Figure 3C); however, as the level of ln(selenium) increased, the risk of COPD decreased in males (Figure 3D). We did not observe this relationship in females (Figure 3E,F).

## 4. Discussion

In our research, the high concentration of copper was positively correlated with COPD in males in the general US population using a multiple linear regression and restricted cubic spline modeling. While the risk of a male suffering from COPD decreased with the increase in selenium concentration (*p* = 0.008 for overall association). Studies have shown that copper is an important cofactor for some enzymatic reactions [9,31], and its mechanism may be related to Lysyl oxidase-like 2 (LOXL 2) [31,32,33]. LOXL 2 is a copper-dependent amine oxidase, which can activate lung fibroblasts through the TGF-β/Smad pathway, leading to pulmonary fibrosis, which in turn leads to impaired lung function. Selenium is related to the cellular antioxidant defense mechanism. Oxidative stress and chronic inflammation are important features in the pathogenesis of COPD [21]. Selenium is a cofactor of glutathione peroxidase, which can protect the human body from cell membrane damage mediated by reactive oxygen species and free radicals [34,35,36,37].

Selenium, cadmium, and lead are enriched in lung tissue [38], but copper, manganese, and zinc have not been reported. A study has shown that the median concentrations of lead, copper, manganese, selenium, copper, and zinc in the lung were 0.072, 0.026, 0.058, 0.11, 1.10, and 10.7 µg/g wet weight (ppm), respectively, among eight individuals in the age range 43 to 72 years [38]. The biological half-life of copper from the diet is 13–33 days, with bilary excretion being the major route of elimination [39]. Experiments of inhaled selenious acid and selenium metal aerosols in beagle dogs showed that the long-term component of the whole-body retention function for both inhaled aerosols had a half-life of about 34 days and accounted for about 20% of the initial selenium dose, and urine was the major route of excretion, accounting for 70 to 80% of the excreted selenium [40]. Therefore, one-time measures in serum of copper and blood of selenium are reflective of recent exposures.

The recommended intake of copper is 900 µg/day according to the Food and Nutrition Board (FNB) of the Institute of Medicine of the National Academy of Sciences [41]. The main dietary sources of copper are shellfish, seeds, nuts, offal, wheat bran cereals, whole grain products, and chocolate [41,42]. Although in our study, the concentration of the copper in the COPD group (124.30 µg/dL) was higher than the healthy group (114.25 µg/dL), they did not exceed the normal range (63.5–158.9 µg/dL) [43]. This is consistent with the results of Cen Jiang et al. [44]. Pearson’s research showed that copper had a strong negative correlation with lung function, and the higher serum copper levels reduce the forced expiratory volume in 1 s [45].

We found that copper only increased the risk of COPD in males, not females, but the serum copper in females (126.60 µg/dL) was higher than that in males (105.10 µg/dL) (*p* < 0.001) (Table S1). It might be the difference in sex hormones between males and females. Copper can activate lung fibroblasts through LOXL 2 activation of the TGF-β/Smad pathway, leading to pulmonary fibrosis, which in turn causes COPD [31,32,33]. Estrogen can inhibit the proliferation of fibroblasts through the Raf1-ERK-MAPK pathway [46,47]. The protective effect of estrogen might explain the sex-specific difference to copper. Therefore, based on our study, we suggest that the standard for serum copper concentration should be lowed and established according to gender.

The selenium intake of Americans exceeds 100 µg/d, which is much higher than the recommended intake (55 µg/d) [48] because of the high concentration of selenium in the soil. In addition, 18–19% of adults and children also use dietary supplements containing selenium [49]. The levels of selenium in the healthy group (192.75 µg/L) and COPD group (189.26 µg/L) were higher than normal concentrations (136.7 µg/L) [48]. Selenium is a cofactor of glutathione peroxidase, which can protect the human body from cell membrane damage mediated by reactive oxygen species and free radicals [34,35,36,37]. Wei Feng et al. [50] found that ln(selenium) has a positive linear relationship with the increase in lung function, but the COPD risk was not covered in the study. An RCT proved that selenium combined with vitamin C can alleviate the deterioration of COPD [51]. Our results also showed that selenium can reduce the risk of COPD.

There are some limitations to our study. First, because it was a cross-sectional study, the evidence for causality was not strong. Second, the data came from the NHANES, and it cannot represent the whole situation in the world and it needs to be verified in other populations. Third, there is no professional diagnosis of COPD by a doctor in NHANES. Although the definition of COPD depended on the participants’ answer to “Has a doctor told you have COPD, emphysema and chronic bronchitis?”, which is consistent with the previous studies about COPD using NHANES data, it might cause some bias in our studies.

In the 2015–2016 NHANES data, only copper and selenium exposure were related to the COPD in US male population. It is necessary to conduct in-depth research to verify this result and to investigate its potential mechanism. More research is expected to explore the relationship between metals and COPD, and the standard for serum copper concentration should establish by gender.

## 5. Conclusions

In our research, the high concentration of copper increased the COPD risk in males in the general US population using the multiple linear regression and restricted cubic spline modeling. While the risk of male suffering from COPD decreased with the increasing selenium level, we did not find associations between copper, selenium and the risk of COPD in female. We hope that there will be more research exploring the relationship between metals and COPD, and based on our study, we hope that the standard for serum copper concentration should be lowed and established according to gender.

## Figures and Tables

**Table 1:** Demographic and socio-behavioral characteristics, metals level, and COPD disease status of the study population (N (%)).

Variables	Healthy	COPD	*p*-Value
	(N = 1292)	(N = 107)	
Age	48.81 ± 17.43 ^a^	58.53 ± 16.19 ^a^	<0.001 ***
Sex			0.828
Male	642 (49.69)	52 (48.60)	
Female	650 (50.31)	55 (51.40)	
Race			<0.001 ***
Mexican American	233 (18.03)	8 (7.48)	
Other Hispanic	167 (12.93)	20 (78.69)	
Non-Hispanic White	442 (34.21)	60 (56.07)	
Non-Hispanic Black	252 (19.50)	16 (14.95)	
Other Race	198 (15.33)	3 (2.80)	
BMI (kg/m^2^)			0.019 *
<18.5	18 (1.39)	1 (0.93)	
18.5–25	325 (25.15)	16 (14.95)	
25-30	437 (33.82)	32 (29.91)	
≥30	512 (39.63)	58 (54.21)	
Education			0.025 *
Less than 9th grade	134 (10.37)	17 (15.89)	
9–11 grade	142 (10.99)	15 (14.02)	
High school graduate	293 (22.68)	26 (24.30)	
Some college/AA degree	379 (29.33)	35 (32.71)	
College graduate	344 (26.63)	14 (13.08)	
Ratio of Family Income to Poverty			0.001 **
Under standard level	1071 (82.89)	102 (95.33)	
Above standard level	221 (17.11)	5 (4.67)	
Smoking			<0.001 ***
Current smoking	751 (58.13)	31 (28.97)	
Non-smoking	234 (18.11)	38 (35.51)	
Former smoking	307 (23.76)	38 (35.51)	
Secondhand smoke exposure			0.013 **
Yes	947 (75.39)	69 (64.49)	
No	318 (24.61)	38 (35.51)	
Serum cotinine (ng/mL)	0.03 (0.01, 4.08) ^b^	0.18 (0.01, 227) ^b^	<0.001 ***
Drinking			0.052
Yes	899 (69.58)	84 (78.50)	
No	393 (30.42)	23 (21.50)	
Hypertension			<0.001 ***
Yes	428 (33.13)	67 (62.62)	
No	864 (66.87)	40 (37.38)	
Diabetes			<0.001 *
Yes	198 (15.33)	35 (32.71)	
No	1094 (84.67)	72 (67.29)	
Blood Pb (µg/dL)	0.93 (0.59, 1.49) ^b^	1.20 (0.75, 2.02) ^b^	<0.001 ***
Tertile 1 (0.05–0.71)	445 (34.44)	23 (21.50)	
Tertile 2 (0.72–1.32)	431 (33.36)	34 (31.78)	
Tertile 3 (1.33–23.51)	416 (32.20)	50 (46.73)	
Blood Cd (µg/L)	0.29 (0.18, 0.50) ^b^	0.41 (0.23, 0.93) ^b^	<0.001 ***
Tertile 1 (0.07–0.22)	458 (35.45)	26 (24.30)	
Tertile 2 (0.23–0.42)	426 (32.97)	28 (26.17)	
Tertile 3 (0.43–6.37)	408 (31.58)	53 (49.53)	
Blood Mn (µg/L)	9.52 (7.72, 11.91) ^b^	9.72 (7.61, 11.83) ^b^	0.862
Tertile 1 (2.31–8.30)	427 (33.05)	40 (37.38)	
Tertile 2 (8.31–10.96)	439 (33.98)	27 (25.23)	
Tertile 3 (10.97–56.56)	426 (32.97)	40 (37.38)	
Blood Se (µg/L)	192.75 (178.99, 207.38) ^b^	189.26 (176.96, 201.26) ^b^	0.070
Tertile 1 (119.87–183.22)	427 (33.05)	40 (37.38)	
Tertile 2 (183.23–201.75)	423 (32.74)	43 (40.19)	
Tertile 3 (201.76–318.33)	442 (34.21)	24 (22.43)	
Serum Cu (µg/dL)	114.25 (99.15, 133.10) ^b^	124.30 (110.40, 145.00) ^b^	<0.001 ***
Tertile 1 (52.90–105.00)	454 (35.14)	18 (16.82)	
Tertile 2 (105.01–126.60)	425 (32.89)	38 (35.51)	
Tertile 3 (126.61–306.60)	413 (31.97)	51 (47.66)	
Serum Zn (µg/dL)	79.90 (69.80, 89.90) ^b^	80. 90 (72.50, 91.10) ^b^	0.408
Tertile 1 (31.40–73.70)	442 (34.21)	27 (25.23)	
Tertile 2 (73.71–86.40)	416 (32.20)	48 (44.86)	
Tertile 3 (86.41–139.10)	434 (33.59)	32 (29.91)	

**Note:** *, *p* < 0.05; **, *p* < 0.01; ***, *p* < 0.001. ^a^ The continuous variables were represented by mean ± SD. ^b^ Non-normally distributed continuous variables were represented by IQR: P50(P25, P75).

**Table 2:** Risk of COPD associated with level of metals in different genders.

variables	Male			Female		
	OR	*p*	95% CI	OR	*p*	95% CI
Pb						
T1	1.00	1.00	1.00	1.00	1.00	1.00
T2	1.20	0.752	0.38–3.79	0.78	0.522	0.36–1.68
T3	1.70	0.362	0.54–5.31	0.68	0.384	0.28–1.63
*p* for trend	0.279			0.388		
Cd						
T1	1.00	1.00	1.00	1.00	1.00	1.00
T2	0.65	0.378	0.26–1.68	0.80	0.576	0.37–1.74
T3	1.64	0.235	0.73–3.69	0.48	0.110	0.19–1.18
*p* for trend	0.160			0.109		
Mn						
T1	1.00	1.00	1.00	1.00	1.00	1.00
T2	0.90	0.796	0.41–1.99	0.66	0.278	0.31–1.40
T3	1.74	0.122	0.86–3.50	0.85	0.654	0.41–1.74
*p* for trend	0.147			0.696		
Se						
T1	1.00	1.00	1.00	1.00	1.00	1.00
T2	0.86	0.683	0.43–1.75	1.79	0.092	0.91–3.53
T3	0.48	0.078	0.22–1.08	0.83	0.633	0.38–1.81
*p* for trend	0.081			0.806		
Cu						
T1	1.00	1.00	1.00	1.00	1.00	1.00
T2	1.65	0.200	0.77–3.52	2.53	0.113	0.80–7.96
T3	3.31	0.004 **	1.47–7.44	2.38	0.128	0.78–7.28
*p* for trend	0.004 **			0.247		
Zn						
T1	1.00	1.00	1.00	1.00	1.00	1.00
T2	1.43	0.332	0.69–2.96	1.81	0.124	0.85–3.86
T3	0.72	0.423	0.32–1.62	1.70	0.195	0.76–3.80
*p* for trend	0.420			0.212		

**Note:** ** *p* < 0.01. The models were adjusted for age, smoke, ratio of family income to poverty, BMI, sex, race education, drink, second-hand smoke exposure, serum cotinine, hypertension, and diabetes.

## Data Availability

All data in the article can be downloaded for free in the NHANES database from https://www.cdc.gov/nchs/nhanes/ (accessed on 1 May 2021).

## Supplementary Materials

The following supporting information can be downloaded at: https://www.mdpi.com/article/10.3390/ijerph19042085/s1. Figure S1: Distribution of exposure variables, Table S1: Level of metals in male and female.
