# Supplementary material for: The Relationship between Metal Exposure and Chronic Obstructive Pulmonary Disease in the General US Population: NHANES 2015–2016
Source: Int J Environ Res Public Health. 2022 Feb 13;19(4):2085. doi: 10.3390/ijerph19042085 (PMC8871875; doi:10.3390/ijerph19042085)
Supplement: Supplementary file 1 [file ijerph-19-02085-s001.zip › ijerph-1568319-supplementary.pdf]

Supplementary Material

Table S1. Levels of metals in male and female.

Variables	Male	Female	*p*-value
All group			
Pb (mg/dL)	1.13(0.71,1.83)	0.79(0.52,1.32)	<0.001 ***
Cd (mg/L)	0.27(0.17,0.49)	0.34(0.20,0.56)	<0.001 ***
Mn (mg/L)	8.77(7.28,11.07)	10.26(8.37,12.82)	<0.001 ***
Se (mg/L)	194.89(181.57,209.63)	190.35(175.90,204.94)	<0.001 ***
Cu (mg/dL)	105.10(93.30,118.20)	126.60(110.80,146.90)	<0.001 ***
Zn (mg/dL)	81.50(71.10,91.90)	78.50(69.00,88.30)	<0.001 ***
Healthy group			
Pb (mg/dL)	1.11(0.7,1.73)	0.79(0.51,1.30)	<0.001 ***
Cd (mg/L)	0.27(0.17,0.45)	0.34(0.20,0.55)	<0.001 ***
Mn (mg/L)	8.71(7.27,10.95)	10.27(8.44,12.90)	<0.001 ***
Se (mg/L)	195.52(182.35,210.48)	190.46(175.65,205.20)	<0.001 ***
Cu (mg/dL)	104.25(92.60,117.00)	126.15(110.70,146.90)	<0.001 ***
Zn (mg/dL)	81.55(71.10,92.00)	77.80(68.90,88.10)	<0.001 ***
COPD			
Pb (mg/dL)	1.64(1.13,2.30)	0.92(0.60,1.59)	<0.001 ***
Cd (mg/L)	0.59(0.25,1.00)	0.38(0.22,0.71)	0.159
Mn (mg/L)	9.83(7.51,12.01)	9.71(7.62,11.93)	0.884
Se (mg/L)	189.46(178.85,201.26)	188.38(178.21,201.26)	0.598
Cu (mg/dL)	117.15(103.60,136.55)	131.60(116.70,148.10)	0.005 **
Zn (mg/dL)	80.45(71.05,87.60)	81.60(75.00,91.70)	0.369

Note: **, $p < 0.01$; ***, $p < 0.001$.

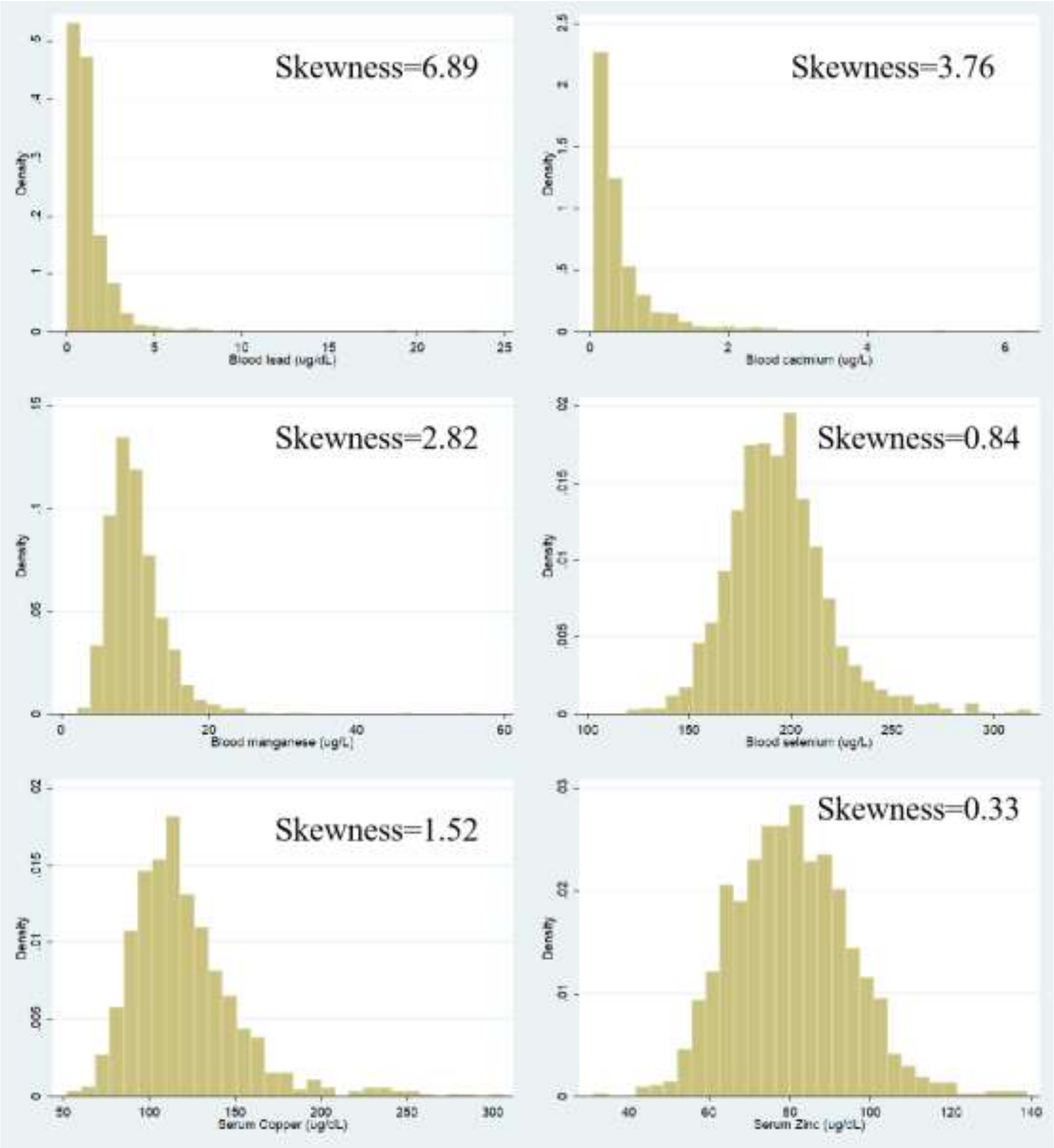

Figure S1. Distribution of exposure variables.
